# A Catalog of Regulatory Sequences for Trait Gene for the Genome Editing of Wheat

**DOI:** 10.3389/fpls.2016.01504

**Published:** 2016-10-06

**Authors:** Szabolcs Makai, László Tamás, Angéla Juhász

**Affiliations:** ^1^Department of Applied Genomics, Centre for Agricultural Research, Hungarian Academy of SciencesMartonvásár, Hungary; ^2^Department of Plant Physiology and Molecular Biology, Eötvös Loránd UniversityBudapest, Hungary; ^3^State Agriculture Biotechnology Centre, School of Veterinary and Life Sciences, Murdoch University, PerthWA, USA

**Keywords:** wheat, glutenins, genetic regulation, crop improvement, promoter diversity of wheat wild relatives, ideotype for genome editing

## Abstract

Wheat has been cultivated for 10000 years and ever since the origin of hexaploid wheat it has been exempt from natural selection. Instead, it was under the constant selective pressure of human agriculture from harvest to sowing during every year, producing a vast array of varieties. Wheat has been adopted globally, accumulating variation for genes involved in yield traits, environmental adaptation and resistance. However, one small but important part of the wheat genome has hardly changed: the regulatory regions of both the x- and y-type high molecular weight glutenin subunit (HMW-GS) genes, which are alone responsible for approximately 12% of the grain protein content. The phylogeny of the HMW-GS regulatory regions of the *Triticeae* demonstrates that a genetic bottleneck may have led to its decreased diversity during domestication and the subsequent cultivation. It has also highlighted the fact that the wild relatives of wheat may offer an unexploited genetic resource for the regulatory region of these genes. Significant research efforts have been made in the public sector and by international agencies, using wild crosses to exploit the available genetic variation, and as a result synthetic hexaploids are now being utilized by a number of breeding companies. However, a newly emerging tool of genome editing provides significantly improved efficiency in exploiting the natural variation in HMW-GS genes and incorporating this into elite cultivars and breeding lines. Recent advancement in the understanding of the regulation of these genes underlines the needs for an overview of the regulatory elements for genome editing purposes.

## Background

Hexaploid wheat only exists in a cultivated form, and it is derived from a cross between the cultivated *Triticum turgidum* subsp. *dicoccum* and a wild goat grass (*Aegilops tauschii*). In one possible scenario its progenitors were as follows: *Triticum urartu* – A genome, *Aegilops speltoides* – B genome and *Aegilops tauschii* – D genome.

The progenitors of wheat acquired many morphological and physiological improvements, such as loss of seed shattering, increased yield, decreased chemical, and morphological defenses, loss of seed dormancy, uniformity in germination and growth phenology, and erect growth in order to facilitate increased plant density in crop fields. These are collectively referred to as a ‘domestication syndrome’ (Allaby, 2014). Further traits were acquired during the cultivation process such as diversification in grain starch composition, adaptation to different climates and latitudes, and the decrease in grain protein to carbohydrate ratio (Harlan et al., 1973; Harlan, 1992; Dubcovsky and Dvorak, 2007). Among the domesticated crops only hexaploid wheat went through speciation, while all other plants retained their genetic relations to their wild types (Harlan et al., 1973).

One detrimental consequence of domestication is the decreased genetic diversity of genes related to domestication syndromes. This genetic restriction, also called *genetic bottleneck*, may restrict the possibilities of the breeder. Indeed, haplotype analysis of high molecular weight glutenin subunit (HMW GS) genes in bread wheat reported less genetic diversity than their wild counterparts, which is mainly due to the genetic bottleneck caused by human selection processes (Giles and Brown, 2006; Dong et al., 2013). Interestingly, in the Central and Southern Asian regions HMW GS diversity is slightly higher than in other territories, which is due to the fact that hexaploidization event of wheat happened in this region (Terasawa et al., 2010) and many ancient landrace populations have remained in cultivation by small farmers. At present, the only ways to increase diversity are to develop synthetic wheat utilizing the genetic variability of the genome donors, or by backcrossing with wild relatives that treasures a yet unchartered genetic depository (Charmet, 2011). However, in light of recent publications, genome editing is expected to revolutionize crop breeding (Belhaj et al., 2013; Upadhyay et al., 2013; Budak et al., 2015; Schiml and Puchta, 2016). Indeed, genome editing was already successfully used for wheat to develop heritable resistance to powdery mildew (Wang et al., 2014). Refined genome editing with reduced off-target mutation will accelerate the adoption of important genes in breeding programs. It seems that knock-out mutants by non-homologous end-joining pathways may have limited use (Svitashev et al., 2015), therefore small insertions or targeted gene replacement based upon genome editing hold greater potential for crop improvements (Voytas and Gao, 2014). However, for targeted gene replacement mutants site specificity is crucial, and this can be successfully achieved by inhibiting non-homologous end joining (NHEJ) and increasing the efficiency of homology-directed repair (HDR). One such solution was already tested in animal production using homolog recombination stimulant RS-1 (Song et al., 2016). In the case of plants, Zhao et al. (2016) recently published an alternative strategy for gene replacement where they achieved a 0.8% success rate. Although application of genome editing is currently not without challenges and difficulties, it is widely anticipated that these can be overcome by further development of the technology as reviewed recently by Schaeffer and Nakata (2015). Based on this assumption, it is essential to have a clear understanding of the genetic options offered as templates by the wild relatives (Charmet, 2011) in order to obtain novel genetic variability for cultivated wheat.

Regulatory regions are one of the major contributors to forming novel traits. Genome wide association studies highlighted the importance of non-coding genomic regions in phenotypic variation in plants (Cubillos et al., 2012). According to Olsen and Wendel (2013), most of the domesticated traits were gained via mutations in either the coding or the promoter region of the genes (Olsen and Wendel, 2013). In case of maize, the gene *teosinte branched1 (tb1)* is reported to be responsible for a major domestication syndrome, and a polymorphism analysis showed that the reduction of diversity was the most severe in the 5′-UTR (untranslated region) (Wang et al., 1999). They concluded that due to the abrupt nature of this reduction, recombination allowed the uncoupling of the coding region from the 5′-UTR. Doebley et al. (2006) reported that changes in development and morphology are related to mutations in transcriptional regulator genes. One noticeable mutation is the Q allele that may have appeared in durum or bread wheat first (Simons et al., 2006). This gene is an AP2 type transcription factor and its product, the Q allele, increases the number of flowers per spikelet thus increasing the sink capacity (Simons et al., 2006).

Well-characterized and important trait genes of wheat are the HMW GS genes which are major contributors to the end-use quality of wheat flour (Shewry et al., 2002). Wheat is a primary protein source for human consumption, and has been extensively studied, but not detailed here. The genes are located on the *Glu-1* locus on the chromosome 1 of the three homoeologous genomes of hexaploid wheat. Due to a duplication event, each locus contains two paralogous glutenin genes named as x- and y-type subunits (Kong et al., 2004). Their coding regions have been thoroughly analyzed and compared. The three *Glu-1* loci of hexaploid wheat have different level of sequence variation, indicating a different history of evolution before hexaploidization (Ciaffi et al., 1998; Rodríguez-Quijano et al., 2001; Gu et al., 2004; Giles and Brown, 2006; Jiang et al., 2012; Dong et al., 2013). The HMW GS genes are found in all species of the *Triticeae* tribe.

Our earlier study reported that the regulatory regions of *Glu-1* genes have a conserved structure of seven *cis*-regulatory modules (CRM) including the proximal basal promoter region (Makai et al., 2014b). The motif compositions of regulatory regions vary across the x- and y-type pairs as well as across the homeologous genes, causing variation in expression activity. However, the x to y ratio of HMW GS proteins is currently sub-optimal from the perspective of end-use. Experiments with transgenic wheat showed that overexpression of y-type HMW GS genes had a more favorable effect on the mixing properties of the dough than overexpression of x-type (Blechl et al., 2007; León et al., 2009). Titration experiments concluded that an appropriate x:y ratio is needed for dough extensibility (Butow et al., 2003; Anderson and Bekes, 2011). Reconstitution experiments using rice flour as a base demonstrated that 1:1 ratio of x- and y-type HMW GS gave the largest effect (Oszvald et al., 2011). Therefore, any attempt to improve this ratio by increasing the activity of the y-type HMW GS genes may positively affect the protein composition of the wheat grain and consequently the bread-making quality of the dough. However, the low allelic differences detected for these regions (Makai et al., 2014a) have severely restricted genetic gains from breeding to date. Genetic variation may be increased by introducing novel elements from related wild species. However, altering such a tiny part of the genomes by traditional crossing is nearly impossible, which leaves genome editing as the most viable technology to address the problem. Here we propose a possible strategy for gene/promoter replacement via studying the phylogeny of the regulatory regions of HMW GS genes of *Triticeae* and exploring the potential advantages of a new type of promoter.

## Phylogeny of the Regulatory Regions of HMW GS Genes

The high similarity between the regulatory region of the homeologous HMW GS (also referred as *Glu-1*) genes raises many questions: (i) What genetic variability is available in the wild species? (ii) How and when were the paralogs (x and y type) separated? (iii) Which gene was the original copy and how has the evolution of the regulatory regions changed during the duplication? (iv) What are the signs of functional shift that are usually characteristic of duplicated genes?

**FIGURE 1:**
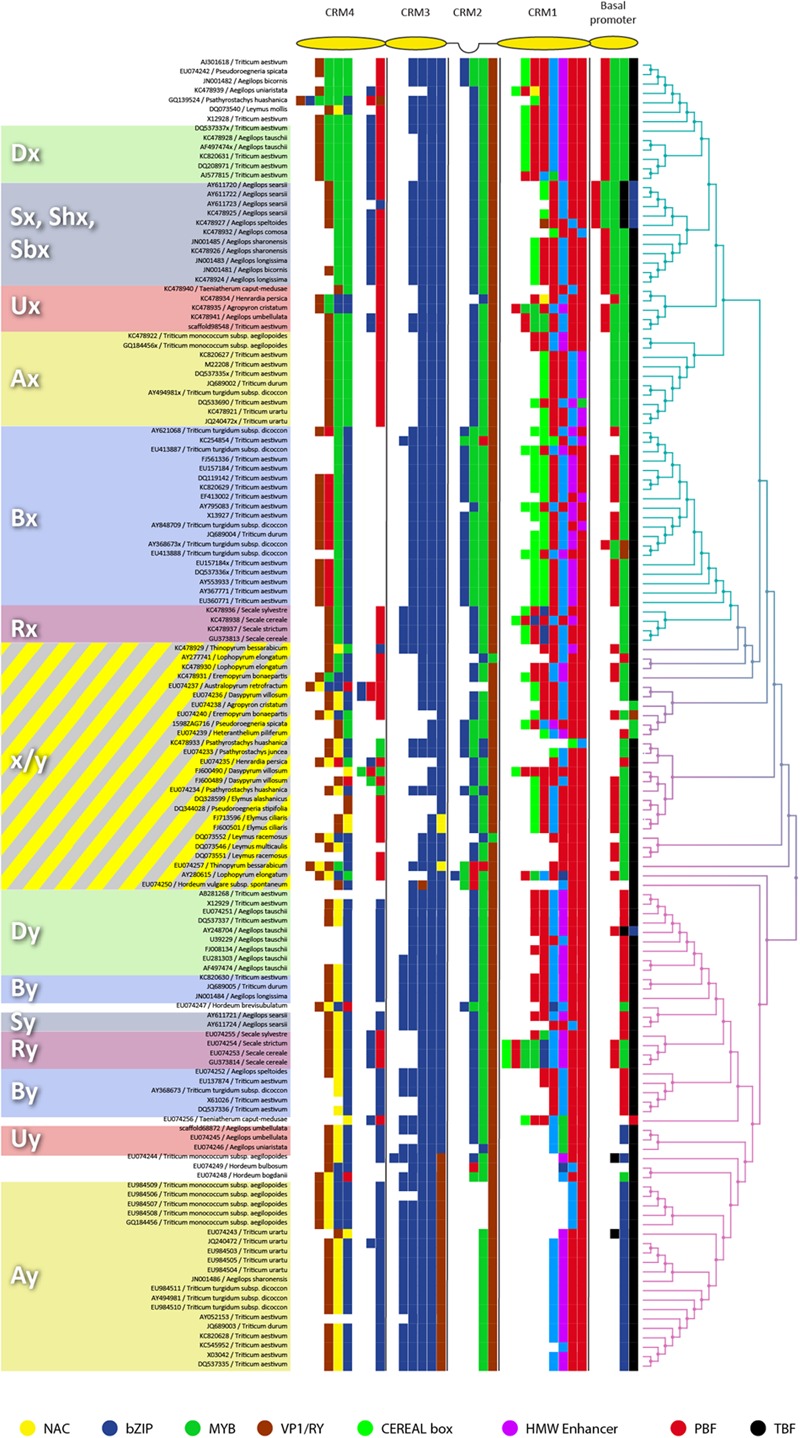
**The phylogenetic tree based on the 900 bp long 5′-UTR of high molecular weight glutenin subunit (HMW GS) genes of 139 *Triticeae* species.** All publicly available (at the time of writing) sequences were used. The sequence data was obtained from the NCBI nucleotide database. The tree was build using the Neighbor-joining method of the MEGA6 software (Tamura et al., 2013). The blue branch marks the x-type promoters, the magenta branch the y-types. The transient gradient represents the hybrid type promoters. Typically, species belonging to the genome E are placed on this transient part. Condensed promoter view is shown where only the motifs and their order are presented. The accession and the species are written in small letters. The motifs to generate the promoter profiles are as follows (in regex format): bZIP (including SPA) - (TGACGT| [GA]TG[AT]G[TA]CAT| GTCAT| GATGACGTGTC); MYB - (TAACAA| AACAAA| AAACCA); NAC – CATGTG; VP1 – CATGCA; TBF - TATA[AT]A[AT][AG]; PBF - TG[GC]A{3,4}[GC]; CEREAL Box - GACATG[GC]TTAGAAG[TC][TA]TTGAGTG; HMW Enhancer - [TG]TTTT[GAC][GC](AAA| CAA| AA)GC[TA]CCAATTGCTCCTT[GA]CTTATCCAGCT.

A phylogenetic study was used to compare 139 regulatory sequences (or promoters) of HMW GS from nearly 40 species of the *Triticeae* tribe. These species represented the A, B, D, E, H, S, R, and V genomes. The sequences were first aligned, and then a phylogenetic tree was built (**Figure 1**). The tree has two main branches separating the x- and y-type promoter paralogs. The branches are marked with different colors. The figure also presents a *condensed* view of the regulatory sequences marking only the binding sites (BS) of transcription factors (TF). These binding sites are known for their involvement in the regulation of HMW GS genes. The x-type promoters carry the unique CEREAL box motif and MYB BS at their basal promoter regions. The y-type promoters are missing these features but carry a NAC binding site at their CRM4 region that was lost in the x-type promoters. The condensed promoter view offers a graphical representation of the changes in the promoters. Many motifs disappear by the growing distance from the middle to form the typical x- and y-type promoter profiles at the outer parts of the tree. The sequences at the middle part of the tree (including *Dasypyrum villosum, Leymus* and *Elymus* species) contain features of both x- and y-type promoters therefore we refer to them as *hybrid* type promoters.

Earlier expression analysis demonstrated that the paralogous HMW GS genes had distinct expression profiles (Hurkman et al., 2013; Makai et al., 2014b). The x-type genes had a peak during the grain-filling stage while the y-type genes showed a gradually increasing but generally lower activity. In addition, a co-expression based focused network analysis identified two distinct gene regulatory networks (GRN) for the paralogs (Makai et al., 2015). Both the transition to grain filling and the transition from grain-filling to maturation are promoted by abscisic acid (ABA) (Finkelstein et al., 2002; Kanno et al., 2010). Based on the relative abundance of ABA-related TFs (LEC1, TaABI5) in their GRN, it is assumed that the x-type HMW GS genes are closely linked to ABA. In contrast, the gene composition of the GRN and their gradually increasing expression profile suggest that y-type HMW GS genes are less dependent on ABA and probably are under a NAC/NAM type regulation (Shinozaki and Yamaguchi-Shinozaki, 2007; Makai, 2015). It is likely that the promoters of the ancient, pre-duplication HMW GS genes were similar to the hybrid type promoters identified in the V and E genome species. Consequently, following duplication, and most importantly during domestication and cultivation, the x-type promoter became closely aligned to the regulatory pathways driving the grain filling stage of the developing endosperm. In other words, the x-type genes may become the primary sink for the nitrogen supply during grain filling. In addition, the coding region of the two paralogs carry distinctive conserved features (number and distribution of cysteine residues, repetitive regions) (Shewry and Tatham, 1997; Shewry et al., 2009), which may indicate a yet unknown functional difference in storage accumulation or protein *packing* or trafficking. This is in concert with the view that after duplication the expression patterns and/or the function of genes are shifted apart (Li et al., 2005; Gibbs and Donohue, 2014).

**FIGURE 2:**
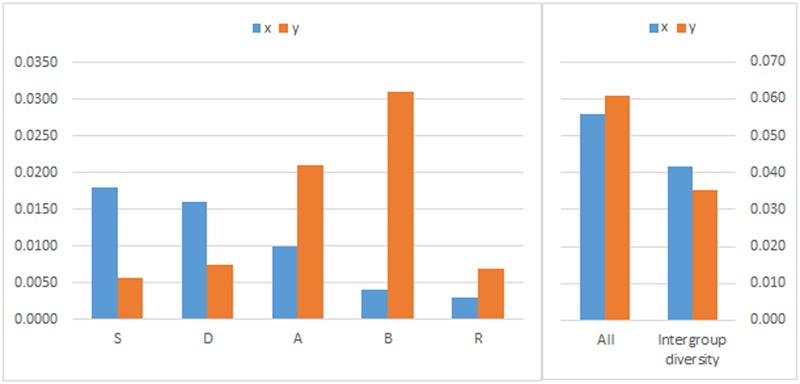
**The divergence of the promoters of *Glu-1* genes in wheat.** Analyses were conducted using the Maximum Composite Likelihood model. All positions containing gaps and missing data were eliminated. Evolutionary analyses were conducted in MEGA6.

In order to have an overall view of the diversity of the regulatory regions of HMW GS genes, the sequences were grouped by genomes and the values of divergence were calculated between the groups (**Figure 2**). In the case of the A, B, and R genomes the x-type promoters had lower divergence values, while in the case of the S and D genomes it was the y-type promoters. The reasons for these biased differences in diversity are unknown. The tetraploid species (AB) were longer exposed to the selective pressure of mankind (Feuillet and Muehlbauer, 2009) and their end-use purpose was (and widely still is) different compared to the hexaploid wheat. This may have driven the AB species toward a different gene set. The calculation of in-between group divergence gave a slightly higher value for the y-type promoters. Although this difference is small, it raises an interesting question: Could the lower level of expression hide any effect on the phenotype, thus easing the grip of selection that led to higher diversity? More sequence data would likely help to find an answer.

## Nature Versus Breeding

The phylogeny of the regulatory regions of HMW GS genes offers a view on how domestication and subsequent breeding have influenced these regions. The analysis demonstrates that the emergence of variation identified in the promoter profiles of hexaploid wheat precedes its evolution, which suggests that breeding has had no influence on the polymorphisms of regulatory regions of HMW GS genes. It is very likely that breeding has affected the activity of GRNs that directly or indirectly are involved with grain protein content (GPC). Many studies have reported the importance of TFs and their polymorphisms as a contributor to improved quality. The haplotype analysis of storage protein activator (SPA) genes has presented evidence for such correlations (Ravel et al., 2009). Genetic mapping in barley has indicated that prolamin-binding factor (PBF) is associated with grain protein content (Haseneyer et al., 2010). Later, the PBF-B was shown to be in linkage disequilibrium with GPC related markers in wheat (Plessis et al., 2013). Both SPA and PBF are directly interacting with the regulatory region of HMW GS genes. Furthermore, the GPC-B1 quality trait locus was shown to be related to NAC transcription factor (Uauy et al., 2006).

The hybrid type promoters are unique to the relatives of wheat. This suggests that *natural* selection had a preference for this type of promoter. Since GRNs are conserved across species (Pires et al., 2013; Shrestha et al., 2014), it is most probable that the two distinct GRNs identified in hexaploid wheat are also present in the wild relatives of wheat. Consequently, the storage protein genes of the wild relatives may be controlled by two interwoven regulatory circuits: an ABA-dependent and an ABA-independent circuit (Agarwal et al., 2011; Nakashima et al., 2014). While the exact mechanism is still unclear, the high drought tolerance of wild species may offer a clue for one possible advantage of this regulatory strategy (Molnár et al., 2004; Akpınar et al., 2013; Dulai et al., 2014). Considering the involvement of ABA in the signal transduction pathway of drought (Zhang et al., 2006), it is hard not to think of these **hybrid promoters** as a “bullet-proof” regulatory region that **can secure the needs of germination in a broad spectrum of environmental conditions.** High levels of ABA (in response to drought) may promote an early transition from grain filling to maturation stage (Chen et al., 2013), however, the ABA-independent pathway would keep up the transcription of these storage compounds in dry conditions.

In conclusion, the hexaploid (bread) wheat may have finely tuned regulatory mechanisms controlling the expression of HMW GS genes that produces high yield in optimal conditions. In contrast, the regulatory regions of HMW GS of the wild relative seem to be better adapted to environmental changes. With regard to the increasing amount of evidence suggesting the role of TF in yield (Sreenivasulu and Schnurbusch, 2012) and with regard to the restricted diversity of regulatory regions of HMW GS genes, the question arises: could any change in the promoters of HMW GS genes make a better use of the transcriptional mechanisms present in the developing endosperm of the hexaploid wheat? Or more specifically, would the hybrid type promoters of the wild relatives coupled with the GRNs of the hexaploid wheat stabilize the grain yield without a loss in protein content even in drought conditions? In the light of our current knowledge, the answer is most probably positive. The wild relatives of wheat offer a diverse catalog of regulatory regions and naturally occurring mutations. Amongst them, wild relatives possessing hybrid type promoters may be of particular interest. Their binding site composition and distribution may offer a way to rewire the regulation of the lower expressing y-type genes to make the most out of the two GRNs. The hybrid type promoters of *Pseudoroegneria spicata, Heteranthelium piliferum*, or *Thinopyrum bessarabicum* may take full advantage of both GRNs. They could potentially stabilize GPC in a wider range of environmental conditions, thus narrowing the gap between the expected and potential quality.

However, a precisely targeted gene replacement of the regulatory regions of HMW GS via genome editing currently faces many challenges. One is due to the high homology between the paralogs and homeologs. To overcome this, the precise sequence data of the targeted region and its homeologs and paralogs of the chosen wheat genotype must be known and guide RNA(s) should be designed accordingly. Other challenges may be the low efficiency of HDR mediated gene replacement and the low frequency of successful transgenic events in plants as recently reviewed by Altpeter et al. (2016). However, even with all these issues, genome editing holds a great potential to overcome the effect of linkage drag in backcross breeding, via directly replacing, deleting, or inserting genetic material to the desired locus.

## Author Contributions

SM conceived the original idea and suggested the proposed view. SM, LT, and AJ wrote the manuscript.

## Conflict of Interest Statement

The authors declare that the research was conducted in the absence of any commercial or financial relationships that could be construed as a potential conflict of interest.
